# Safety and effectiveness of transcervical radiofrequency ablation for uterine fibroids in patients with obesity: a retrospective cohort study

**DOI:** 10.1007/s00404-025-08265-3

**Published:** 2026-01-30

**Authors:** Elvin Piriyev, Mariam Sadikova, Angelika Dieter, Sven Schiermeier, Stefan Peter Renner, Thomas Römer

**Affiliations:** 1 University Witten-Herdecke, Witten, Germany; 2 Department of Obstetrics and Gynecology, Academic Hospital Cologne Weyertal, Weyertal 76, 50931 Cologne, Germany; 3 University of Cologne, Cologne, Germany; 4 Department of Gynecology and Obstetrics, Boeblingen Clinic, Hospital Sindelfingen-Böblingen, Böblingen, Germany

**Keywords:** Obesity, Uterine fibroids, TFA, Radiofrequency, AUB, Hypermenorrhea

## Abstract

**Key message:**

Transcervical radiofrequency ablation is a low-risk, uterus-preserving option for symptomatic fibroids in women with obesity with significant improvement of bleeding disorder, including ≥ 40 kg/m^2^. Obesity should not preclude offering TFA.

**Objective:**

To evaluate the safety and effectiveness of transcervical radiofrequency ablation (TFA) for uterine fibroids in women with obesity.

**Methods:**

Retrospective multicenter cohort at two German Fibroid Centers. From 574 consecutive TFA cases, we included patients with BMI ≥ 30 kg/m^2^ and ≥ 6-month follow-up; those with incomplete data were excluded. Fibroids were characterized by ultrasound. TFA (Sonata®) was performed per instructions for use. Outcomes were perioperative complications and patient-reported improvement in abnormal uterine bleeding (AUB).

**Results:**

Sixty patients were analyzed (age 43.59 ± 6.52 years; BMI 35.72 ± 6.72 kg/m^2^). Mean operative and ablation times were 33.65 and 9.91 min, respectively. One intraoperative bleeding event (1.7%) was controlled with a balloon catheter; no postoperative complications occurred. Mean follow-up was 17.08 months (6–54). Overall, 42/60 (70.0%) reported AUB improvement. By BMI category: 30–34.9 kg/m^2^ 25/39 (64.1%), 35–39.9 kg/m^2^ 5/7 (71.4%), ≥ 40 kg/m^2^ 12/14 (85.7%) (p = 0.3168). Considering the initial assessment, 48/60 (80.0%) improved; six later recurred, yielding 42/60 (70.0%) at last follow-up.

**Conclusion:**

TFA showed a very low complication rate and clinically meaningful bleeding improvement in women with obesity, with comparable outcomes across BMI strata, including ≥ 40 kg/m^2^. Obesity is not a barrier to safe, effective TFA. Prospective, BMI-stratified studies with validated bleeding measures and objective endpoints are warranted.

The present study aimed to investigate the effectiveness and safety of combined approach transcervical fibroid ablation in patients with obesity.

## Introduction

Uterine fibroids are the most common gynecologic tumors, with an estimated prevalence ranging from 4.5% to 68.6% depending on age, ethnicity, and diagnostic method [1]. Prevalence is age-dependent, peaking at 65.2% among women aged 46–50 years [2]. Despite their high prevalence, the pathogenesis and risk factors of this benign disease are not fully understood [3]. Fibroids cause a spectrum of symptoms; heavy menstrual bleeding (HMB) is most frequent (40–54%), followed by dysmenorrhea and lower abdominal pain [4, 5]. In up to 48% of cases, fibroids are associated with severe HMB and anemia [6]. Hysterectomy remains the most common treatment for symptomatic fibroids, and fibroids are still the leading indication for this procedure [7–10]. Fibroids account for 40–60% of all hysterectomies and for approximately 30% of hysterectomies among women aged 18–44 years [11]. The International Federation of Gynecology and Obstetrics (FIGO) classification provides a standardized system for describing fibroid location, which—together with size and number—guides prognosis and treatment selection [10, 12].

In parallel, obesity has risen steadily over the past two decades. Globally, an estimated 16% of adults were living with obesity in 2022, with continued upward trends. German surveillance likewise shows increases across sex and education strata through 2023, reaching 18% among women [13, 14]. Because obesity is highly prevalent in the reproductive-age population, it frequently coexists with symptomatic fibroids. Obesity adds clinical and technical complexity to management: compared with open surgery, minimally invasive approaches generally reduce wound complications, venous thromboembolism, and length of stay in patients with obesity, yet access to and completion of laparoscopy can be challenging at higher BMI classes. These considerations motivate uterus-preserving options that avoid abdominal incisions and peritoneal entry when feasible [15].

Transcervical radiofrequency ablation (TFA) with intrauterine ultrasound guidance (Sonata® system) is an incisionless therapy that treats a broad range of FIGO fibroid types via targeted thermal ablation [16–19]. Some studies have demonstrated durable symptom improvement, quality-of-life gains, uterine preservation, and low perioperative complication rates over 2–5 years, supporting the safety and effectiveness of TFA in appropriately selected patients [19–21]. However, evidence specifically addressing patients with obesity remains limited. Published TFA cohorts rarely report BMI-stratified outcomes, and only small case series include women with high BMI, leaving an important knowledge gap for counseling and perioperative planning in this large patient group [17].

To address this gap, we conducted a retrospective cohort study evaluating the safety and effectiveness of transcervical radiofrequency ablation for uterine fibroids in patients with obesity**.** We hypothesized that TFA would maintain a favorable perioperative safety profile and yield clinically meaningful improvements in symptoms and quality of life irrespective of obesity status.

## Methods and materials

**Fig. 1:**
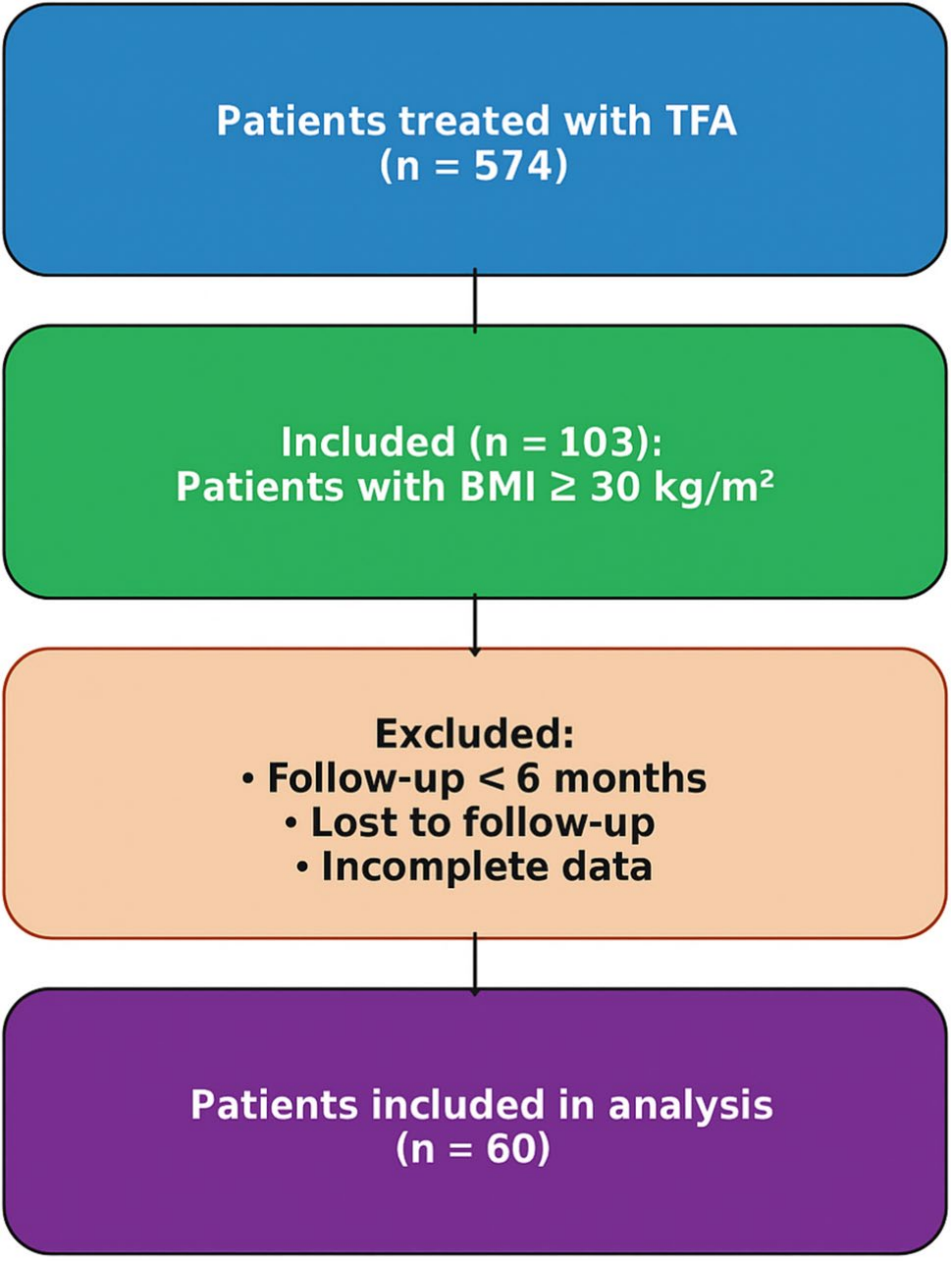
Flow of participants from the total TFA cohort to the analysis set

This retrospective, multicenter cohort study evaluated patients with uterine fibroids treated with transcervical radiofrequency ablation (TFA) using the Sonata® system at two Fibroid Centers of Excellence in Germany: Academic Hospital Cologne Weyertal and Hospital Böblingen. A total of 574 consecutive patients were screened. For the present analysis, we included only patients with hypermenorrhea and obesity, defined according to the World Health Organization (WHO) criteria [13]. Patients were excluded if baseline data were incomplete, if they were lost to follow-up, or if follow-up was shorter than 6 months (Fig. 1).

Uterine fibroids were identified and measured by transvaginal and/or transabdominal ultrasound, as clinically appropriate. The size (maximum diameter) and number of fibroids were recorded.

All procedures were performed with the Sonata® system according to the manufacturer’s instructions for use. After intrauterine placement of the device, fibroids were localized using the integrated ultrasound probe. Treatment planning included definition of the intended ablation zone (“red zone”) and safety margins (“green zone”). Following confirmation of the safety zone, the electrodes were deployed and thermal ablation was initiated once the target temperature of 105 °C was reached.

Perioperative safety was assessed by recording intraoperative and postoperative complications. Patients receive standardized discharge instructions to return or contact us immediately during the first 4 postoperative weeks if any warning symptoms occur (fever, increasing pelvic pain, foul-smelling discharge, or heavy bleeding). Effectiveness was assessed at a minimum of 6 months after TFA by evaluating changes in bleeding symptoms and patient satisfaction. Follow-up was conducted either by telephone interview or during the patient’s most recent clinic visit. Patients provided a subjective assessment of improvement in bleeding symptoms and their overall satisfaction with treatment.

Body mass index (BMI) was calculated as weight in kilograms divided by height in meters squared (kg/m^2^), and categorized according to World Health Organization (WHO) criteria [13]:

- BMI 30–34.9: obesity I°
- BMI 35–39.9: obesity II°
- BMI ≥ 40: obesity III°

### Statistical analysis

Statistical analyses were performed using IBM SPSS Statistics for Windows, Version 25.0 (IBM Corp., Armonk, NY, USA). Continuous variables are presented as mean (standard deviation) and categorical variables as counts (percentages). Where applicable, comparisons between subgroups were conducted using Chi-Square test for categorical data and independent-samples t-tests for continuous data. A two-sided p-value < 0.05 was considered statistically significant.

## Results

Sixty patients were included. The mean age was 43.59 years (SD 6.52; range 30–59). The mean BMI was 35.72 kg/m^2^ (SD 6.72; range 30–53). Age distribution and BMI category are showed in Table 1.

**Table 1:** Demographic and clinical characteristics

Characteristics	Value
Patients	
N	60 (100%)
Age, mean (SD), range (years)	43.59 (6.52); 30–59
Age categories (years)	
30–35	9 (15%)
36–40	10 (16.7%)
41–45	16 (26.7%)
46–50	16 (26.7%)
51–55	7 (11.7%)
> 55	2 (3.3%)
Body mass index (BMI)	
BMI, mean (SD), range (kg/m^2^)	35.72 (6.72); 30–53
BMI categories (kg/m^2^)	
30–34,9	39 (65%)
35–39,9	7 (11.7%)
≥ 40	14 (23.3%)
Fibroid characteristics	
Total fibroid number	114
Fibroid size, mean (SD), range (cm)	4.17 (2.14); 1–10
Fibroids per patient (n patients)	
1 Fibroid	28 (46.7%)
2 Fibroids	19 (31.7%)
3 Fibroids	7 (11.7%)
> 3 Fibroids	6 (10%)
FIGO Classification of Fibroids (n = 106 fibroids)*	
FIGO 2	12 (11.3%)
FIGO 3	18 (17%)
FIGO 4	18 (17%)
FIGO 5	28 (25.9%)
FIGO 6	7 (6.6%)
FIGO 2–5	23 (21.7%)
n.a	8 (7.5%)

^*^Information about FIGO classification was available in 106 fibroids

Across the cohort, 114 fibroids were documented. The mean largest fibroid diameter was 4.17 cm (SD 2.14; range 1–10). Most patients had one fibroid 28 (46.7%). FIGO type was available for 106 fibroids. Most common fibroids were FIGO 5 and FIGO 2–5, 25.9% and 21.7% respectively. In 8 cases data on FIGO classification was not available (Table 1).

Table 2 illustrates the intra- and postoperative characteristics.

**Table 2:** Perioperative outcomes and complications

Outcome	Value	Notes
Operative duration, mean (SD), range (min)	33.65 (15.98); 16–73	
Ablation time, mean (SD), range (min)	9.91 (7.43); 1.1–40.3	
Intraoperative complications, n (%)	1 (1.7%)	Bleeding; managed with balloon catheter
Postoperative complications, n (%)	0 (0.0%)	

Study population: n = 60 patients

Mean follow-up was 17.08 months. Only 12 (20%) patients had follow-up duration less than 12 months. Overall, 42/60 patients (70.0%) reported improvement in uterine bleeding. By BMI category, improvement was reported by 25/39 (64.1%) in the 30–34.9 kg/m^2^ group, 5/7 (71.4%) in the 35–39.9 kg/m^2^ group, and 12/14 (85.7%) in the ≥ 40 kg/m^2^ group, with no significant difference across categories (p = 0.3168) (Table 3).

**Table 3:** Follow-up and improvement of uterine bleeding

Characteristics		P value
Follow-up duration mean (SD), range (months)	17.08 (11.32); 6–54	
Follow-up duration	n/N (%)	
6–12 months	32/60 (53.3%)	
13–24 months	18/60 (30%)	
> 24 months	10/60 (16.7%)	
Improvement of uterine bleeding*		
Overall	42/60 (70%)	
By BMI category		
30–34,9 kg/m^2^	25/39 (64.1%)	0.3168
35–39,9 kg/m^2^	5/7 (71.4%)	
≥ 40 kg/m^2^	12/14 (85.7%)	

^*^Improvement refers to patient-reported improvement in bleeding symptoms

Among the 18 patients with no improvement of symptoms at the time of follow-up, 6 (33.3%) reported an initial improvement in symptoms followed by recurrent or new symptoms during follow-up, yielding an initial improvement rate of 48/60 (80.0%). The time to first recurrence after TFA was a mean (SD) of 18.7 (12.34) months (range 9–41). Five of the six underwent subsequent surgical management: three had hysterectomy (recurrence after 12, 17, and 24 months of initial improvement), and two underwent laparoscopic and abdominal myomectomy (recurrence after 9 months of initial improvement). In one patient with recurrence after 41 months, no additional intervention was recorded by the end of follow-up (total follow-up 53 months).

## Discussion

In this multicenter cohort of women with obesity undergoing TFA, we observed a favorable safety profile with meaningful symptom improvement. Procedural complications were rare—one intraoperative bleeding event managed with a balloon catheter and no postoperative complications—and patient-reported initial bleeding improved in 80% and decreased to 70% at a mean follow-up of 17.08 months. Improvement did not differ significantly across BMI strata (30–34.9; 35–39.9; ≥ 40 kg/m^2^), suggesting that higher BMI alone does not diminish clinical benefit after TFA. Although subgroup numbers are small, the numerically highest response in the ≥ 40-kg/m^2^ group (85.7%) supports the feasibility of TFA even in class III obesity.

Obesity is positively associated with the risk of uterine fibroids. A recent meta-analysis reported a modest but significant increase in odds with higher BMI (OR 1.19; 95% CI 1.09–1.29) [22]. Management options span medical therapy to hysterectomy [5, 7, 11, 12, 16–21]. Fibroids remain the leading indication for hysterectomy [7–10], yet in a survey of nearly 1,000 women with fibroids, 80% wished to avoid invasive treatment and nearly half wished to avoid hysterectomy [10]. Treatment selection should integrate FIGO type, fibroid size and number, symptom severity, reproductive plans, comorbidities/contraindications, and patient preference.

Bipolar operative hysteroscopy is the most commonly used approach for submucosal fibroids [12, 23]. However, large (> 3 cm), multiple (n > 3), and FIGO type-2 lesions can challenge hysteroscopic management; in such cases, 20–50% of patients may require at least one additional session for complete treatment [12, 23–25]. While intraoperative risks with hysteroscopy are generally low [23], postoperative intrauterine adhesions have been reported in 10–37.5% after resection of a single fibroid and up to 45% with multiple fibroids [12, 23, 26]. Because operative hysteroscopy is limited to submucosal disease, patients with a broader fibroid distribution may benefit from a combined strategy using TFA for intramural/transmural lesions and hysteroscopy for cavity pathology [16, 27].

Laparoscopic myomectomy carries distinct intra- and postoperative risks at several phases—hysterotomy, enucleation, hemostasis, and morcellation—with postoperative issues including hematoma at the hysterotomy site, intrauterine or abdominal adhesions, and rarely parasitic myomas [28–30]. Reported complication rates vary: in 654 laparoscopic myomectomies (mean fibroid diameter 5.3 cm), intraoperative complications occurred in 2.6% and postoperative in 5.7% [28], whereas a larger series of 2.050 resections noted an overall rate of 11.1% [31]. Several authors describe rising complication rates with laparoscopic enucleation, potentially reflecting wider adoption among surgeons with less experience in suturing, dissection, or electromechanical morcellation [28, 32, 33]. Obesity further increases risk after abdominal myomectomy: in a contemporary cohort of 27.387 cases, postoperative complications occurred in 11.4% overall and rose to 16.1% in class-III obesity (P < 0.001). In general, complication rate in class II and III obesity was higher compared to normal BMI [34]. For laparoscopy, higher BMI (≥ 30 kg/m^2^) has been associated with longer operative time (OR 1.7; 95% CI 0.8–3.7; p = 0.20), indicating a non-significant trend toward increased complexity [35]. Operative efficiency is another consideration. Catanese et al. reported an operative time of 95 min (42–177) for laparoscopic myomectomy in patients with a single fibroid of approximately 6 cm (range 1–7 cm) and a median BMI of 21.6 kg/m^2^ [35]. In our cohort, the mean operative time for TFA was 33.65 min (16–73) (Table 2). Although cross-study comparisons should be interpreted cautiously given differing techniques and populations, TFA appears to be completed in markedly less time (p = 0.0001). A recent study of 206,944 patients undergoing laparoscopic hysterectomy demonstrated that obesity is associated with a higher complication rate and prolonged operative time [36].

In our present study, the majority of patients (70–80%) experienced a significant reduction in abnormal uterine bleeding after TFA, and this outcome appears consistent with published data [16–21, 37]. Notably, patient satisfaction and symptom relief remain high in both our cohort and published studies through the follow-up period, underscoring that TFA offers enduring benefits in managing fibroid-related bleeding. From a safety standpoint, TFA showed an excellent profile in women with obesity. Procedures were short (operative duration 33.65 min; ablation 9.91 min), there was only one intraoperative bleeding event—controlled with an intrauterine balloon—and no postoperative complications were recorded. Importantly, safety and effectiveness were comparable across BMI strata, with no signal of increased perioperative risk in patients with ≥ 40 kg/m^2^ and the highest numerical improvement rate (85.7%) observed in this group. The incisionless, transcervical route likely contributes to this favorable profile by avoiding abdominal entry, trocar placement, pneumoperitoneum, and morcellation—steps that typically magnify wound, anesthetic, and thromboembolic risks in higher BMI. Taken together, our data indicate that obesity is not a barrier to safe, effective TFA and support its use as a uterus-preserving option for patients who wish to avoid laparotomy or prolonged laparoscopy.

Obesity is an important factor to consider in patients with abnormal uterine bleeding, independent of fibroids. Excess body weight is known to increase the baseline risk of heavy menstrual bleeding through hormonal and inflammatory mechanisms. For instance, higher BMI and central obesity are associated with greater odds of experiencing heavy menstrual bleeding even in women without fibroids [38]. Biologically, obesity contributes to a pro-inflammatory environment in the endometrium and can delay the repair of the uterine lining during menstruation, leading to heavier and prolonged bleeding [39]. In the context of TFA, these obesity-related factors could dampen the bleeding improvement achieved by the procedure in some patients. Indeed, our follow-up data revealed that some patients showed initial improvement in bleeding after TFA but then experienced a recurrence of significant bleeding symptoms later. Such patterns may be explained by the underlying obesity-driven AUB tendencies that persist despite successful fibroid ablation. In other words, while TFA effectively addresses fibroid-related bleeding, coexisting risks like obesity and its associated endometrial dysfunction can limit the overall improvement or contribute to relapse of heavy bleeding over time, emphasizing the need for a holistic approach to managing AUB in obese patients.

## Strengths and limitations

This study’s strengths lie in its real-world, multicenter design and its deliberate focus on women with obesity, a group often under-represented in fibroid research. By reporting BMI-stratified outcomes, including patients with ≥ 40 kg/m^2^, and applying a standardized TFA technique, we provide granular, clinically useful data. Consecutive screening from a large TFA database (n = 574) with transparent inclusion/exclusion criteria strengthens internal validity, and follow-up extending to 54 months (mean 17.08 months) offers a meaningful view of durability.

Limitations reflect the retrospective design and sample size. Bleeding improvement was patient-reported rather than captured with a validated instrument (e.g., UFS-QoL/SSS), and we lacked a non-obese comparator and systematic volumetric/sonographic follow-up. Heterogeneous follow-up, partly via telephone, introduces recall bias, and selection factors may limit generalisability.

## Conclusion

BMI ≥ 30 kg/m^2^ should not be a barrier to offering transcervical radiofrequency ablation for symptomatic fibroids. In our two-center cohort, TFA was completed safely with only one intraoperative bleeding event, no postoperative complications, and short operative times; initial rate of symptoms improvement was 80% decreased at a mean 17.08-month follow-up to 70.0% of patients, with comparable benefit across BMI strata and the numerically highest response in those with ≥ 40 kg/m^2^. These findings support TFA as a practical, uterus-preserving option for patients with obesity. Prospective, BMI-stratified studies using validated bleeding metrics, objective hematologic/imaging endpoints, and longer follow-up are warranted to refine selection and quantify durability and reintervention risk.

## Data Availability

No datasets were generated or analysed during the current study.
